# From Molecular Design to Practical Applications: Strategies for Enhancing the Optical and Thermal Performance of Polyimide Films

**DOI:** 10.3390/polym16162315

**Published:** 2024-08-16

**Authors:** Liangrong Li, Wendan Jiang, Xiaozhe Yang, Yundong Meng, Peng Hu, Cheng Huang, Feng Liu

**Affiliations:** 1 Fuzhou Medical School, Nanchang University, Fuzhou 344000, China; ncurong@163.com (L.L.); jd15297829717@163.com (W.J.); 18170835662@163.com (X.Y.); 2 School of Chemistry and Chemical Engineering, Nanchang University, Nanchang 330031, China; 3 Jiangxi Shengyi Technology Co., Ltd., Jiujiang 332005, China; mengyd@syst.com.cn (Y.M.); hup@syst.com.cn (P.H.); huangc@syst.com.cn (C.H.)

**Keywords:** polyimide films, optical transparency, thermal stability, molecular design, structural optimization

## Abstract

Polyimide (PI) films are well recognized for their outstanding chemical resistance, radiation resistance, thermal properties, and mechanical strength, rendering them highly valuable in advanced fields such as aerospace, sophisticated electronic components, and semiconductors. However, improving their optical transparency while maintaining excellent thermal properties remains a significant challenge. This review systematically checks over recent advancements in enhancing the optical and thermal performance of PI films, focusing on various strategies through molecular design. These strategies include optimizing the main chain, side chain, non-coplanar structures, and endcap groups. Rigid and flexible structural characteristics in the proper combination can contribute to the balance thermal stability and optical transparency. Introducing fluorinated substituents and bulky side groups significantly reduces the formation of charge transfer complexes, enhancing both transparency and thermal properties. Non-coplanar structures, such as spiro and cardo configurations, further improve the optical properties while maintaining thermal stability. Future research trends include nanoparticle doping, intrinsic microporous PI polymers, photosensitive polyimides, machine learning-assisted molecular design, and metal coating techniques, which are expected to further enhance the comprehensive optical and thermal performance of PI films and expand their applications in flexible displays, solar cells, and high-performance electronic devices. Overall, systematic molecular design and optimization have significantly improved the optical and thermal performance of PI films, showing broad application prospects. This review aims to provide researchers with valuable references, stimulate more innovative research and applications, and promote the deep integration of PI films into modern technology and industry.

## 1. Introduction

Polyimide (PI) films remain as prominent high-performance polymer materials due to their excellent thermal stability, radiation resistance, and electrical insulation properties. These characteristics are indispensable in advanced fields such as microelectronics, flexible displays, optoelectronic materials, electromagnetic shielding, and new energy components [1,2,3,4,5]. With the rapid development of 5G communications, the Internet of Things, and flexible display technologies, PI films are well recognized to be capable of meeting the stringent requirements for high heat dissipation, dielectric performance, and flexible display technology in electronic products, motivating the rapid expansion of the PI films towards emerging markets [6].

The high-temperature stability of PIs places them at the top of the performance pyramid for polymer materials, making them widely used in cutting-edge high-tech fields [5]. However, as the microelectronics industry moves toward large-scale integration and high-speed operation, the thermal and optical properties of traditional PI films have begun to fall short of the increasingly stringent application requirements [7,8,9]. Therefore, enhancing the optical and thermal performance of PI films through molecular structure design has become a hot research topic. Optimizing the backbone structure, introducing side groups, non-coplanar structures, and end groups of PI can significantly improve optical transparency while maintaining high thermal stability, thus expanding its applications in flexible optoelectronic devices and high-performance electronic components.

**Figure 1:**
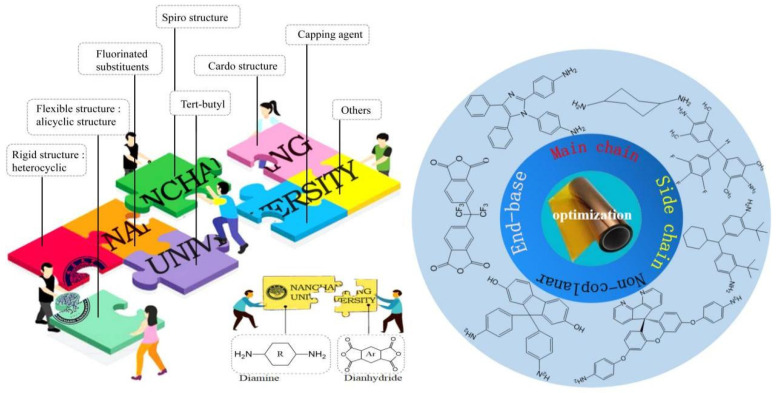
Illustration of PI molecular structure design strategies.

This review aims to systematically summarize and analyze recent research advancements in the molecular design of PI structures, focusing on the strategies to enhance the optical and thermal properties of PI films. Through detailed discussions on methods such as main-chain optimization, side-chain optimization, the introduction of non-coplanar structures, and end-group capping, this paper provides a comprehensive research framework and optimization strategies, as shown in Figure 1. Advantages, disadvantages, and applicability of various strategies were put forward. Additionally, the review elucidates future research directions and discuss the prospects of emerging technologies such as nano-hybrid modification, machine learning-assisted design, and metallization modification. It is expected to provide theoretical guidance and practical references for future research and applications of PI films.

## 2. Overview of PI Film Properties

### 2.1. Basic Characteristics

PI films exhibit a series of excellent properties and are widely applied in many fields. Firstly, PI films possess outstanding thermal stability, chemical durability, and physical properties in high-temperature environments, making them indispensable for high-tech applications. Secondly, PI films demonstrate excellent radiation resistance, capable of withstanding high-energy radiation damage, suitable for space technology and nuclear industries that require high radiation protection. Additionally, PI films have excellent electrical insulation and mechanical properties, providing reliable insulation protection in electrical and electronic equipment while maintaining good mechanical strength and toughness.

### 2.2. Application Fields

As one class of high-performance polymer material, PI films play a significant role in microelectronics, flexible displays [10], optoelectronic materials [11], and energy power components [12] due to their excellent thermal stability, radiation resistance, electrical insulation, and mechanical properties.

In microelectronics, PI films are widely used in manufacturing high-performance electronic components due to their excellent electrical insulation and thermal stability. They serve as electronic packaging materials, circuit board substrates, and insulation layers in flexible circuits [13]. Their high-temperature resistance and mechanical stability ensure the stable performance of electronic devices in harsh working environments.

With the rapid development of flexible display technology, PI films are increasingly used in flexible OLED and curved displays [14]. Their good mechanical flexibility and high transparency make them ideal substrates for flexible displays. PI films can withstand repeated bending and maintain stable optical and mechanical properties in high-temperature processes, which is crucial for the production and use reliability of flexible displays.

In optoelectronic materials, PI films are widely used in optoelectronic devices due to their high transparency and excellent thermal stability [15]. They can be used as substrate and packaging materials, providing good light transmission and environmental durability, thus improving the efficiency and lifespan of the devices. Additionally, PI films can be used in optical waveguides and optical devices due to their excellent optical properties. 

In the energy power field, PI films are valuable as high-voltage insulation materials and heat dissipation materials [16]. Their high heat resistance and electrical insulation properties enable them to provide reliable insulation protection in high-voltage electrical equipment, preventing electric shock and short-circuit accidents. Meanwhile, the excellent thermal conductivity and heat dissipation performance could be obtained by blending nanoparticles with PI films, making them the preferred material for heat dissipation, effectively dissipating heat generated during the operation of electronic devices, ensuring the safe and stable operation of the equipment.

## 3. Strategies for Optimizing Optical and Thermal Performance of PI Films

### 3.1. Main Chain Optimization Strategies

The molecular main chain structure is closely related to the orientation, crystallization, and packing of the PI films [17]. The chain rigidity, flexibility, linearity, and torsion directly affect the PI film’s optical and thermal performance. Therefore, when analyzing the optical and thermal expansion behavior of PI films, the impact of optimizing the PI molecular main chain structure should be prioritized.

The rigid molecular chain of polyimide is the primary reason for the high thermal stability of the PIs. Introducing rigid structures such as heterocycles, polyaromatic, and triptycence structures into the molecular main chain of the PIs can further enhance the conjugation and rigidity of the molecular chain, improving the thermal stability under high-temperature conditions. However, increasing the rigidity of the main chain also increases the optical loss of the PI films, reducing optical transparency. To prepare PI film materials with high transparency, flexible structures and alicyclic structures have been introduced into the molecular main chain to increase chain flexibility, reduce internal rotational barriers, and lower the conjugation degree between aromatic rings, thereby reducing electron mobility and improving transparency.

Currently, extensive research on the rigid structures of PI main chains has been conducted, with common rigid units including benzimidazole and pyridine [18,19], while flexible structures are focused on introducing ether bonds [20], organosilicon [21], and aliphatic structures [22]. Balancing the effects of PI molecular main chain structures on optical and thermal performance to optimize comprehensive properties of PI films still remains a hot topic in future PI material structure–performance research.

#### 3.1.1. Adjusting Main Chain Rigidity

**Figure 2:**
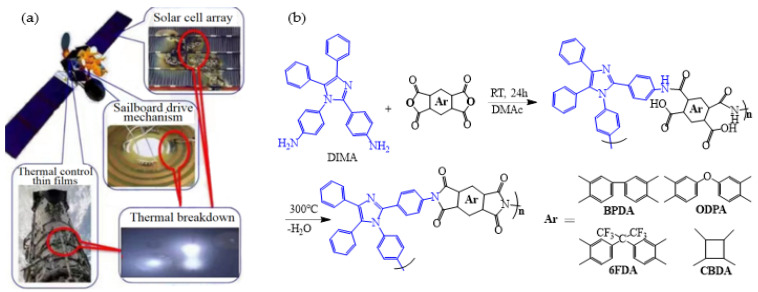
(**a**) Visualization of performance loss PI film applied in aircraft; (**b**) synthesis of PI containing tetraphenyl imidazole structure.

In the field of portable energy materials, using flexible PI films as substrates can significantly improve the flexibility of copper indium gallium selenide (CIGS) solar cells and shorten the efficiency gap between CIGS solar cells and silicon-based solar cells [23]. However, traditional PI films face challenges to be compatible with the high-temperature deposition process of CIGS solar cells and are susceptible to extreme environments such as high vacuum and temperature differences in outer space when applied in aerospace, which imposes higher requirements on the thermal stability of PI films (Figure 2a).

The glass transition temperature (Tg) of PI films is primarily related to the rigidity, stereo-configuration, and intermolecular interactions of the molecular chain. The conjugation between heterocycles and benzene rings can significantly enhance intermolecular forces and improve the thermal stability, thereby breaking the performance limitations of PI materials [24]. Jeong et al. [25] used DIMA, a diamine containing a tetraphenyl imidazole structure, to prepare four PI films (Figure 2b) with biphenyl dianhydride (BPDA), 3,3′,4,4′-oxydiphthalic dianhydride (ODPA), hexafluoroisopropylidene phthalic anhydride (6 FDA), and cyclobutane tetracarboxylic dianhydride (CBDA), respectively. They found that the rigid structure of DIMA significantly restricted the movement of PI molecular chains, improved chain rigidity and regularity, enhanced intermolecular interactions, and significantly improved the thermal stability of the films. In particular, the PI film polymerized with BPDA exhibited highly linear molecular chains, the strongest rigidity, and the best thermal stability, with T_g_ of 367 °C and 5% weight loss temperature (T_d5_) of up to 532 °C in nitrogen atmosphere. The thermal stability of films polymerized with 6 FDA, ODPA, and CBDA, which contain fluorine groups, ether bonds, and alicyclic structures, respectively, was lower, but their optical performance improved. For example, the optical transmittance of DIMA/6 FDA increased to about 96% at 450 nm, and the cutoff wavelength (λ_0_) decreased to 383 nm, still maintaining good mechanical properties. These comprehensive properties provide possibilities for their application in flexible solar cell substrates.

#### 3.1.2. Hydrogen Bond Regulation in the Main Chain

With the rapid increase in demand for flexible displays, there is an urgent need for flexible polymer materials with excellent heat resistance to replace glass substrates. While some commercially available PI films possess outstanding heat resistance and low coefficients of thermal expansion (CTE), further research is needed to reduce CTE values and improve optical transparency for broader applications in flexible display technology. Studies have shown that introducing hydrogen bonds into PI molecular chains can suppress chain mobility and reduce free volume, thereby lowering the in-plane CTE values and of PI films [26,27].

Current research on hydrogen bond regulation primarily focuses on incorporating amide and hydroxyl hydrogen bonds, with amide hydrogen bonds playing more significant parts. The mainstream strategy involves synthesizing diamine or dianhydride monomers containing amide bonds, used to create PI films with amide–amide and amide–imide hydrogen bonds. The regulation effect of amide–amide hydrogen bonds in the main chain is particularly notable, as it enhances the thermal stability and mechanical performance while maintaining optical transparency. Introducing trifluoromethyl groups in the side chain can further enhance hydrogen bonding, cleverly achieving an optimal balance of optical and thermal properties [28,29].

**Figure 3:**
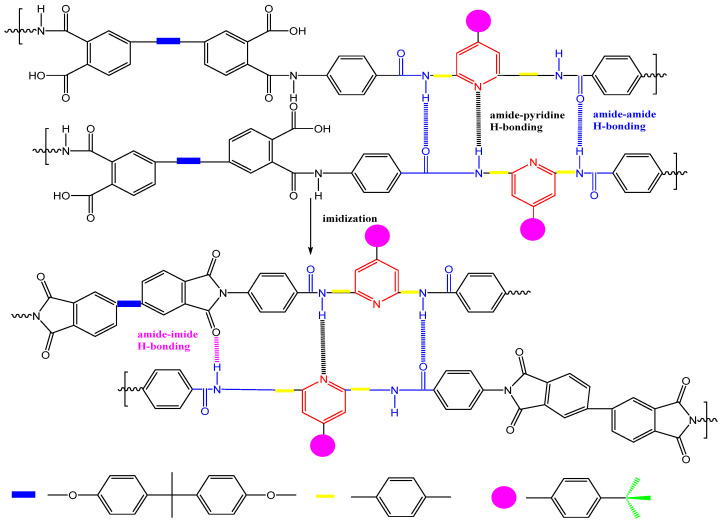
Three types of hydrogen bonding between the interchain NTPA–BPADA segments.

Our research group [30] synthesized a novel diamine monomer, NDPA, containing amide bonds, tert-butyl, and pyridine rings. By copolymerizing NDPA with BPADA and ODA, we prepared a series of PI films and analyzed the impact of forming amide–amide, amide–imide, and amide–pyridine hydrogen bonds on the combined optical and thermal performance (as shown in Figure 3). Introducing tert-butyl and pyridine rings suppressed charge transfer complex formation, offering PI films with high optical transparency, achieving up to 83% transmittance at 500 nm. The presence of appropriately spaced hydrogen bonds did not alter the aggregation state of the polymer matrix. These non-covalent hydrogen bonds effectively increased the rigidity of the PI molecular chains, reducing chain mobility and compensating for the loss of thermal stability and mechanical properties. The films exhibited a maximum tensile strength of 127 MPa and T_g_ ranging from 244 to 298 °C.

In terms of hydroxyl hydrogen bond regulation, our research group [31] also synthesized a novel diamine containing aliphatic amino and hydroxyl groups, then copolymerized it with ODPA and ODA to prepare a series of PI films. The introduction of aliphatic structures improved film transparency, reducing λ_0_ to the 328–370 nm range. The hydroxyl groups contributed to the formation of hydrogen bonds that increased chain cross-linking and rigidity, without significantly compromising thermal properties. Similarly, Yang et al. [32] synthesized PI films using 2,5-bis(4-aminophenyl)pyridine with PMDA and ODA. They found that strong hydrogen bonds formed between hydroxyl groups and nitrogen atoms in heterocycles increased chain entanglement and suppressed segmental motion, enhancing chain packing density, in-plane orientation, and rigidity, thus maintaining high thermal stability. When n(ODA)/n(PRD) was 0:1, the CTE of the PI film decreased to 0.55 ppm/K.

These strategies effectively counteract the negative impact of tert-butyl and alicyclic structures on the thermal and mechanical properties of PI films. PI films with balanced comprehensive properties show promise for use as substrate materials in flexible organic light-emitting diode (OLED) displays and flexible printed circuit boards (FPC). However, a significant challenge remains as the hydrogen bonds could dissociate under harsh service conditions, potentially compromising PI film performance. Therefore, finding methods to enhance the stability of strong hydrogen bonds is crucial for developing ideal flexible substrate PI films with high heat resistance, thermal dimensional stability, and excellent flexibility [33,34].

#### 3.1.3. Incorporation of Alicyclic Structures into the Main Chain

Compared to traditional rigid glass substrates, flexible PI films used as substrates for flexible thin-film solar cells offer advantages such as light weight and flexibility. However, balancing the fundamental properties of thermal resistance, high-temperature dimensional stability, mechanical flexibility, and optical transparency remains a key technical challenge. The issue lies in improving the thermal properties of PI films while enhancing their optical performance. A comprehensive review of transparent, colorless polyimides that balance optical and thermal properties has been provided in two recent articles [35,36], summarizing the related synthesis processes, optimization strategies, and engineering applications.

**Figure 4:**
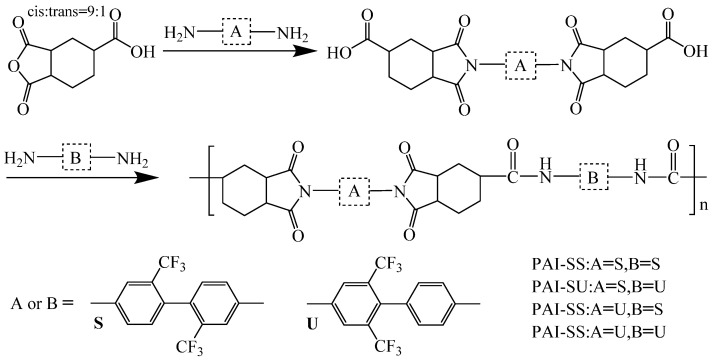
Synthetic scheme of alicyclic PAIs.

Introducing flexible structures improves optical transparency and solubility but sacrifices PI’s inherent thermal properties. Balancing thermal stability and transparency, optimizing the PI molecular structure to enhance strengths while mitigating weaknesses, and developing high-temperature-resistant transparent PI films are ongoing research hotspots. Lee et al. [37] found that designing larger-volume alicyclic structures could weaken intramolecular conjugation and intermolecular CTC interactions, improving optical transparency while retaining good thermal stability. To better balance and optimize the optical and thermal properties of PI films, their research team reacted 1,2,4-cyclohexanetricarboxylic acid with 2,6-bis(trifluoromethyl)benzenamine (U) and 2,2′-bis(trifluoromethyl)benzenamine (S) to introduce amide groups into the PI main chain, synthesizing a series of novel polyamide–imide PI films (PAI), as shown in Figure 4. These PAI films exhibited excellent optical properties, with transmittance close to 90% at 550 nm. By introducing amide groups, the bulky alicyclic flexible units were confined within imide and amide bonds, reducing the adverse effects of flexible structures on thermal stability. The films maintained T_d5_ in N_2_ between 418 and 425.8 °C, and T_g_ above 300 °C.

**Figure 5:**
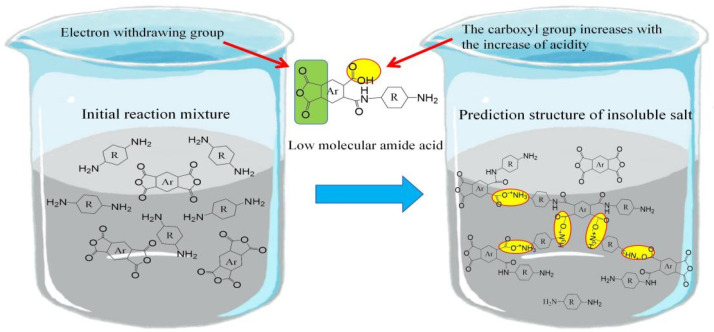
Initial salt formation mechanism of aliphatic PI polymerization.

The introduction of alicyclic flexible units has partially resolved the mutual constraints between the optical transparency and thermal stability of PI. However, studies have shown that at the initial stage of PI film preparation, the strong basicity of alicyclic diamines can lead to the formation of insoluble nylon salts due to acid–base interactions with polyamic acid (PAA). These nylon salts, which exhibit a cross-linked structure, tend to precipitate out during the polymerization of alicyclic diamines and dianhydrides, resulting in poor or even terminated PAA polymerization, as illustrated in Figure 5.

Hasegawa [38], using PMDA and cyclohexane diamine (CHDA) as examples, thoroughly analyzed the mechanism of insoluble nylon salt formation. It was proposed that introducing larger, more twisted, and higher molecular weight alicyclic diamine structures into the main chain can inhibit the formation of these salts. For instance, reacting PMDA with 4,4′-methylenebis(cyclohexane)isophorone diamine, and 2,5(2,6)-bis(aminomethyl)bicyclo[2.2.1]heptane, which possess bulky and twisted structures, demonstrates that such units can reduce the degree of cross-linking in the salts. This allows solvent molecules to penetrate the salt more easily, aiding in its dissolution and ensuring the polymerization of PAA and the performance of the PI film.

The design and selection of alicyclic structural units, and the exploration of flexible structure optimization mechanisms in PI molecules, are crucial in balancing PI’s optical and thermal properties. Introducing flexible structures improves optical transparency and solubility but sacrifices PI’s inherent thermal properties. Managing a balance between thermal stability and transparency, optimizing the PI molecular structure to enhance strengths and mitigate weaknesses, and developing high-temperature-resistant, transparent PI films remain hot topics of research.

In recent years, our research group [39,40,41,42,43,44,45,46,47] has focused on the alicyclic units in the main chain, fluorine-substituted groups in the side chain, and cardo structures in non-coplanar configurations. We have synthesized several bulky alicyclic diamines and non-coplanar alicyclic dianhydride monomers. These studies confirm that the electron-donating and steric effects of bulky alicyclic structures in the PI main chain improve solubility, processability, and hydrophobicity, while non-coplanar bulky structures inhibit charge transfer complex (CTC) formation and reduce intermolecular forces. Based on this, we have explored the synergistic effects of the volume and torsion degree of alicyclic structures, conducting multiple structural characteristic combinations to synthesize novel functional PI materials. Additionally, we have integrated nano-hybrid optimization strategies for PI molecules, studying the impact of PI composite films’ optical and thermal properties and related mechanisms. This aims to expand the application of PI films in image display devices, gas separation membranes, microlenses, and photosensitive materials.

### 3.2. Side-Chain Optimization Strategies

Studies have shown that introducing fluorinated substituents and tert-butyl groups as side chains can effectively produce high optical transparency and light-colored PI films [48]. Introducing fluorinated substituents in the side chain can increase the PI inter-chain spacing, enhancing steric hindrance, reducing chain packing, electron mobility, and CTC formation. The higher the fluorine content, the better the optical transparency of the PI film without compromising thermal stability. However, the preparation of fluorinated PI films involves high costs and complicated synthesis of fluorinated monomers [49,50]. Large-volume side groups like tert-butyl also help cut off CT interactions between electron donors and electron acceptors, increasing chain spacing, reducing CTC formation, and improving transparency and optical properties [51]. However, the synthetic process of new dianhydride and diamine monomers with tert-butyl groups is not yet mature, requiring further research [52].

#### 3.2.1. Fluorinated Substituents

CTC formation is an important cause of color deepening in PI films. CTC formation requires the molecular structure to absorb photon energy and transition to higher energy levels, and the required energy is determined by the energy gap between the lowest unoccupied molecular orbital (LUMO) of the dianhydride and the highest occupied molecular orbital (HOMO) of the diamine. The larger the energy gap, the shorter the wavelength of light absorbed by electrons, resulting in lighter-colored PI films. Therefore, reducing the electron-accepting ability of dianhydrides and the electron-donating ability of diamines can hinder electron transition channels, obtaining light-colored or even colorless transparent PI films [53]. Introducing strongly electronegative fluorine atoms into PI can reduce the HOMO level and increase the energy gap due to their strong electron-withdrawing ability, effectively weakening the CT effect and improving optical transparency.

**Figure 6:**
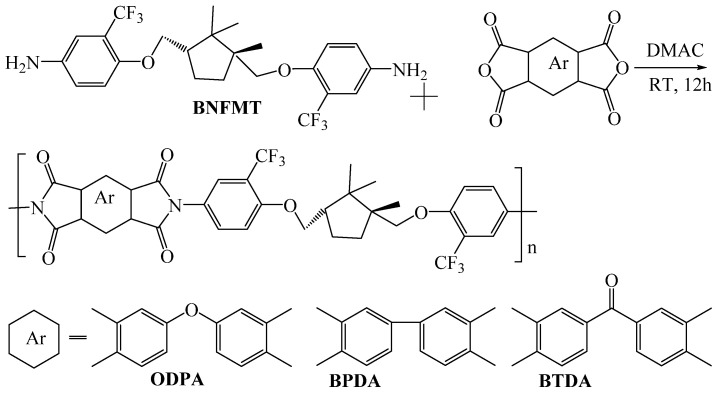
The synthesis of fluorinated PI.

Our research group [54] synthesized a novel fluorinated diamine, BAFMT, using natural camphor as the raw material. Then, a series of new fluorinated cyclic functionalized PI films with ODPA and BPDA was prepared (Figure 6). The study found that the low polarizability of the C-F bond weakened intermolecular forces. The strong electron-withdrawing –CF_3_ group and the bulky trimethylcyclopentyl structure increased steric hindrance and induced effects, effectively reducing interchain charge transfer complexes (CTC) and disrupting dense polymer chain packing. These factors collectively minimized film coloration and improved transparency. Among the films, the PI film made from BAFMT and ODPA exhibited the lowest λ_0_ (341.5 nm) and the highest transmittance at 450 nm (84%).

**Figure 7:**
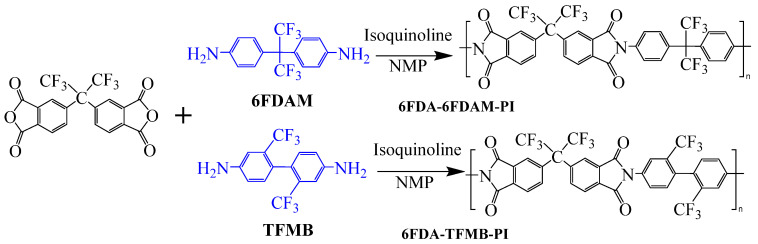
Synthetic schemes of two fluorinated PI.

Min et al. [55] synthesized two highly fluorinated 6 FDA–6 FDAM–PI and 6 FDA– TFMB–PI using 6 FDA with 6 FDAM and TFMB, respectively (Figure 7). The study found that the large-volume –CF_3_ groups weakened chain interactions, and the strong electron-withdrawing effect made electron transfer extremely difficult, inhibiting CTC formation, making PI transparent and colorless in solution and film states. The study also found that these two films had smooth surface morphology and low surface-free energy, with surface-free energies of 36.7 and 34.3 mJ·m^−2^, respectively, and dielectric constants of 2.6 and 2.5. Laboratory-scale flexible organic field-effect transistor (OFET) devices based on transparent flexible FPI materials were prepared by coating this PI solution onto patterned indium tin oxide (ITO) grid electrodes.

**Figure 8:**
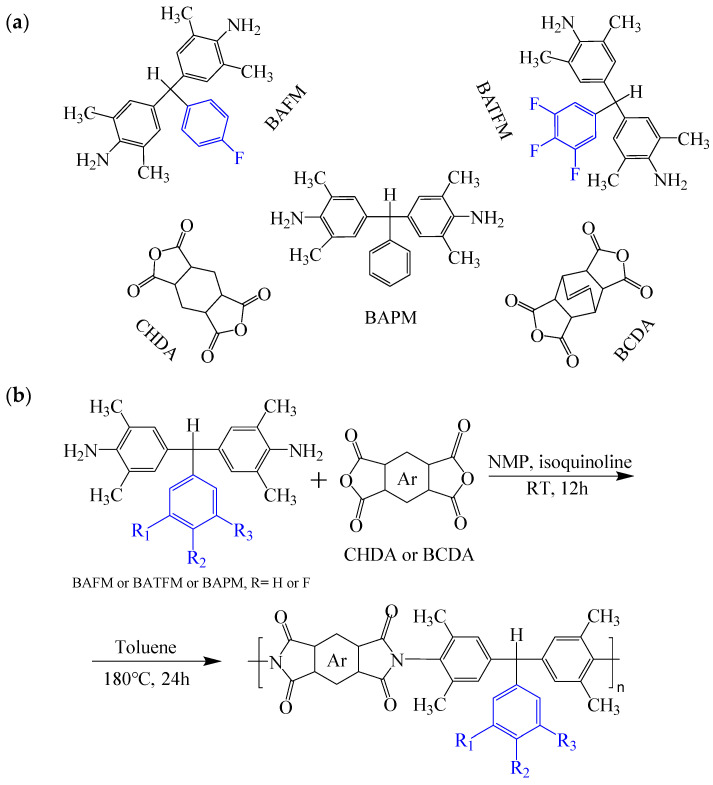
(**a**) Diamine and dianhydride monomers employed in the synthesis of PIs; (**b**) synthesis of semi-aromatic polyimides.

To further study the mechanism of fluorine content on the comprehensive performance of PI films, Liu et al. [56] used non-fluorinated BAPM and three aromatic diamines with fluorine content ranging from 3.4% to 10 wt% (Figure 8a), BAFM and BATFM, to prepare a series of new PI films with CHDA and BCDA (Figure 8b). The study found that the cutoff wavelength λ_0_ of the films ranged from 282 to 286 nm, and the transmittance at 500 nm exceeded 89%. Among them, PIBF–3 and PIBF–6, prepared from BATFMA with the highest fluorine content, had the lowest λ_0_ (282 nm) and the highest transmittance at 500 nm (over 94%). It was realized that there was a significant correlation between fluorine content, energy gap, and optical performance of these semi-aromatic PIs. As the fluorine content of the monomers increased, the HOMO level of the diamine components gradually decreased, the energy gap increased, and the intermolecular and intramolecular charge transfer was effectively inhibited. Compared with the deep yellow commercial Kapton film produced by DuPont, the transparency of this FPI significantly increased. The study also found that this series of films had good thermal properties, with T_d_ in nitrogen ranging from 421 to 492 °C, and T_g_ ranging from 364 to 387 °C, retaining good thermal properties. This study provided a new strategy for designing CPI molecular structures for advanced optoelectronic applications, broadening their applications in flexible display panels, image display substrates, and liquid crystal alignment layers.

#### 3.2.2. Tert-Butyl

**Figure 9:**
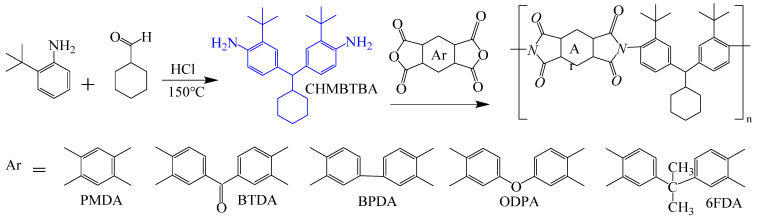
Synthetic route of PIs with bulky pendent groups.

The high cost of raw materials and complex fluorination processes restrict the industrial development of FPIs [57,58]. Therefore, finding other large side groups to replace fluorine and using large free volumes to block electron flow in the molecular chain to improve optical transparency and processability is an essential strategy in optimizing PI molecular structure design. Many studies have found that large-volume tert-butyl side groups can significantly improve the optical transparency and gas separation performance of PI films without compromising thermal stability or processability [59,60]. Wu et al. [61] introduced large amounts of cyclohexyl and tert-butyl units through the Mannich reaction of cyclohexanone and 2-tert-butylaniline, synthesizing diamine CHMBTBA and preparing a series of PI–1~PI–6 films with different aromatic dianhydrides including PMDA, BTDA, BPDA, ODPA, and 6 FDA, as shown in Figure 9. The study suggests that the steric hindrance effect of tert-butyl and cyclohexyl could effectively reduce CT effects and inhibit CTC formation, imparting good transparency to PI films. All PI films exhibited transmittance above 86% in the visible light region, with λ_0_ between 306 and 350 nm. Among them, the transmittance of CHMBTBA/6 FDA film was the best, reaching 90%, with λ_0_ of 306 nm. This series of PI films also showed high thermal stability, with T_d5_ in nitrogen ranging from 487 to 526 °C and T_g_ ranging from 314 to 394 °C.

Incorporating tert-butyl groups into the PI chain can enhance the optical transparency of the film because the tert-butyl groups disrupt the regularity of the chain, increase the intermolecular free volume, and reduce the conjugation degree of the PI molecular structure. However, this modification somewhat compromises the thermal stability of the PI film. When heated above 350 °C, tert-butyl groups in PI films are particularly prone to release, which is a key drawback of using tert-butyl modification to prepare transparent PI films with low thermal resistance. 

**Figure 10:**
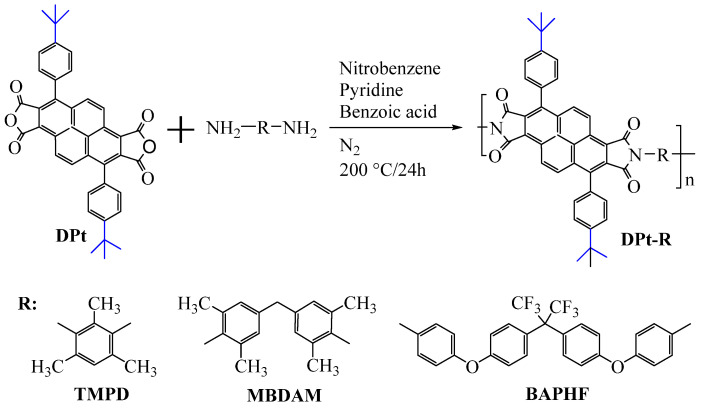
Synthesis of DPt-based polyimides.

To address this, Sulub-Sulub et al. [62] synthesized a novel dianhydride, DPt, containing fused pyrene skeleton with two tert-butyl substituted phenyl groups, and then prepared a series of PI films with TMPD, MBDAM, and BAPHF (Figure 10). The introduction of a large number of polyaromatic structure increased the conjugation degree within the PI structure to maintain the thermal stability of the PI films. The T_d5_ of this series of PI films in N_2_ ranged from 532 °C to 556 °C, with T_g_ values all above 340 °C, and these PI films did not discolor at temperatures above 350 °C. 

Additionally, the incorporation of rigid, bulky tert-butyl groups into the molecular chains effectively inhibited the PI chain packing, breaking chain regularity, increasing intermolecular free volume fraction (FFV), and improving the gas permeability and separation performance of PI films. Among them, the DPt–TMPD film exhibited a CO_2_/CH_4_ selectivity of 9.9 and a CO_2_ permeability of 2035 Barrer at 2 atm and 35 °C, an increase of nearly 27% compared to the DPPD–TMPD film without tert-butyl reported by Santiago-García et al. [63]. This research provided an approach to optimize the design of PIs for high-temperature gas separation membranes.

### 3.3. Non-Coplanar Optimization Strategies

CTC formation between electron-accepting dianhydride and electron-donating diamine monomers within and between molecular chains greatly contributes to deep coloration in PI materials. In addition, the highly conjugated structural characteristics enable easy electron flow within the molecular chain, and the highly planar structure of traditional aromatic PIs further accelerates electron flow, while promoting CTC formation. Therefore, introducing non-coplanar structures into traditional aromatic PI molecular chains can disrupt large π-bonds within the chain, weaken intermolecular interactions, break chain regularity, increase intermolecular free volume, effectively reduce the coplanarity of PI molecular chains, and reduce the optical loss of PI film materials, imparting PI films with high transparency [64]. Currently, research on non-coplanar structural optimization of PI molecules focuses on spiro and cardo structures [65,66]. However, these structural materials also face issues such as plasticization, aging, low chemical stability, poor solubility, and low-temperature resistance in practical applications [67].

#### 3.3.1. Spiro Structure

The spiro structure consists of two or more rings connected through a tetrahedral quaternary carbon atom, acting as the spiro center. The spiro structure constrains the polymer chain to twist at approximately 109° angles at each spiro center [68,69]. Introducing spiro structures into PI molecular chains can effectively limit the density of chain packing and reduce chain interactions, improving the transparency and processability of PI films. Additionally, the spiro structure can maintain a certain rigidity in the PI molecular structure, exhibiting high thermal performance [70].

**Figure 11:**
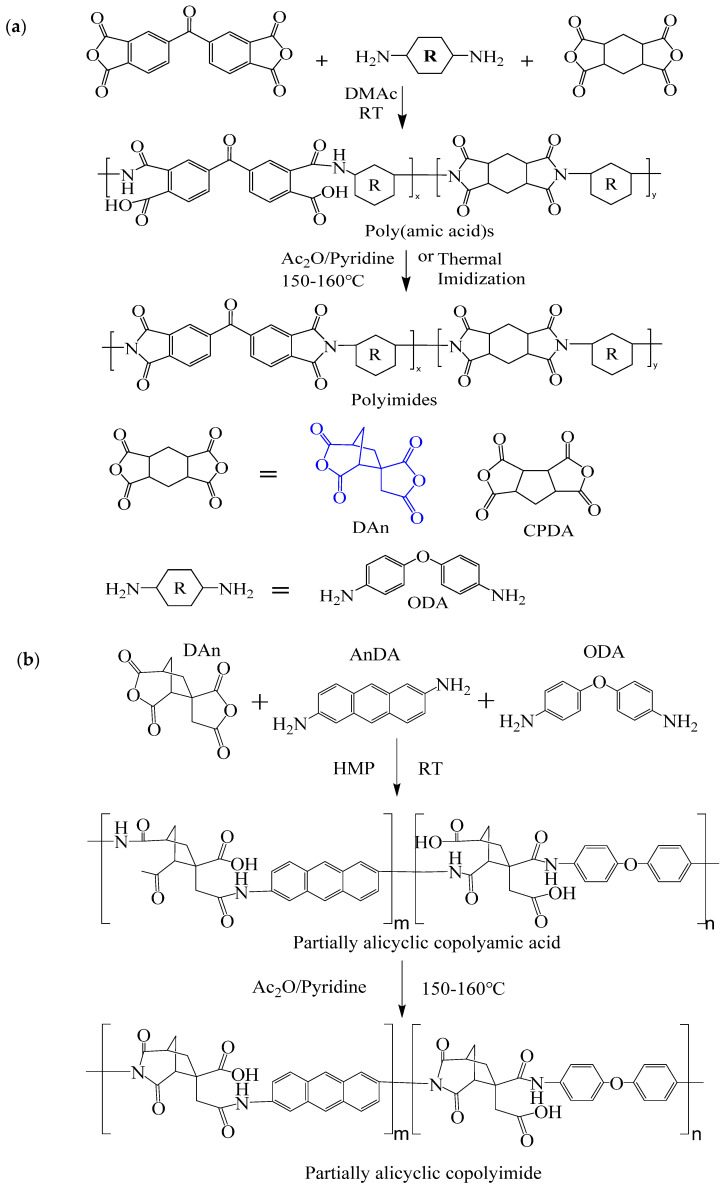
(**a**) Synthesis scheme of PI films containing spiro alicyclic structure; (**b**) synthesis of partially alicyclic polyimides.

Yu et al. [71] prepared a series of PI films by polymerizing spiro-structured dianhydride DAn and alicyclic dianhydride CPDA with diamine ODA (Figure 11a). The study found that the rigid twisted spiro structure disrupted the regularity of PI chains, reducing chain conjugation, while the alicyclic structure further inhibited CTC formation, significantly improving the optical transparency of PI films. The control sample PI–BTDA–100 film appeared yellow–brown, and as the DAn content in the PI structure increased, the film’s color gradually lightened. When n(DAn)/n(ODA) was 0.9:0.1, the PI–DAn–90 film was nearly colorless, with λ_0_ as low as 318 nm and a transmittance of 93% at 400 nm. Additionally, due to the rigid structure of DAn, this series of films exhibited T_d_ above 450 °C and T_g_ exceeding 260 °C, showing good thermal performance.

The research group [72] continued to prepare a series of PI films using DAn and 2,6-diaminoanthracene (AnDA) and 4,4′-oxydianiline (ODA) (Figure 11b). They found that introducing anthracene structures enabled PI films to exhibit ON and OFF conductive states at different voltages, with ODA adjusting the flexibility of PI chains. When n(DAn)/n(AnDA)/n(ODA) was 1:0.7:0.3, the flexible transparent PI-A70-O30 film was obtained. When applied in an ITO/PI/ITO/glass-structured digital memory, it exhibited high transmittance (about 90%) in the 400–800 nm wavelength range and had bipolar resistive switching behavior with stable retention time of 104 s. This series of studies provided ideas for developing transparent memory devices and controlling polymer storage performance.

**Figure 12:**
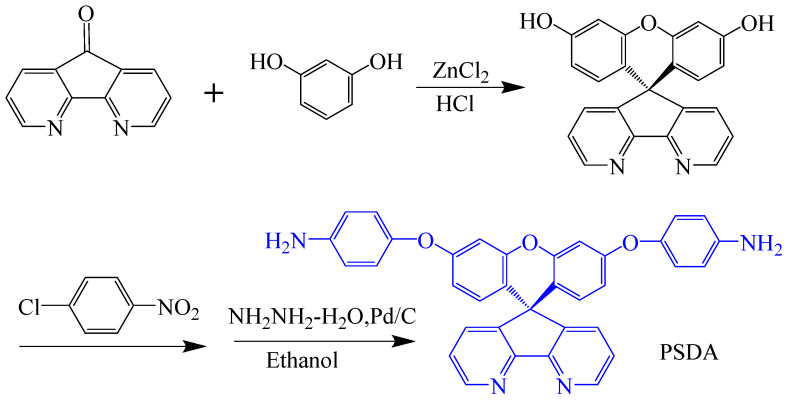
Synthetic scheme of diamine containing rigid spiro aromatic 4,5-dinitrogen fluorene structure.

Li et al. [73] synthesized a new diamine monomer containing 4,5-diazafluorene–9-one (PSDA) through a three-step process, and prepared a series of films with PMDA, BPDA, BTDA, ODPA, and 6 FDA (Figure 12). The study found that the central spiro–4,5-diazafluorene group structure in this new diamine significantly reduced the formation of CTCs between electron donors and acceptors, reducing film optical loss, with λ_0_ ranging from 317 to 415 nm, and transmittance in the visible light region between 67% and 83%, significantly better than commercial Kapton PI films. Additionally, the spiro–4,5–diazafluorene substituent in the molecule maintained the rigidity of the PI backbone, with T_d10_ in nitrogen above 560 °C and T_g_ ranging from 338 to 432 °C, showing good optical transparency and thermal stability.

However, the optical performance of PI films is relatively poor because the rigidity of the spiro structure together with the highly rigid phenyl groups in PMDA cause excessive rigidity and reduce optical performance. Introducing spiro structural units into PI molecular chains and regulating the conjugation of PI molecules should be a feasible strategy for balancing the optical and thermal performance of PI films.

#### 3.3.2. Cardo Structure

**Figure 13:**
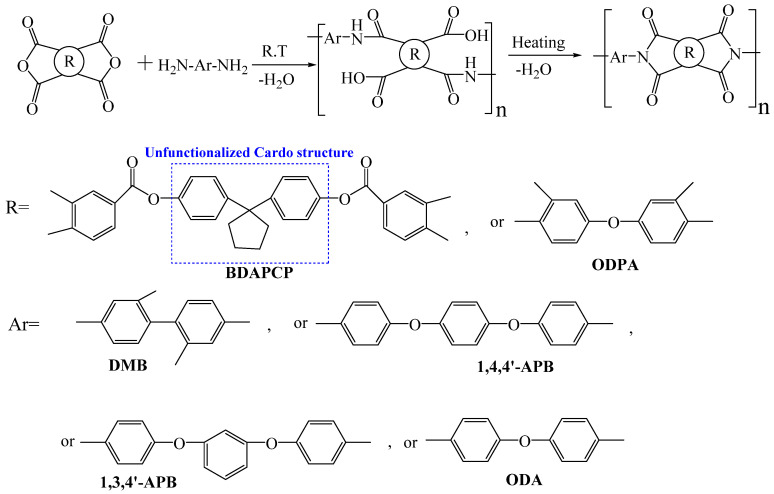
Synthetic scheme of PIs with cardo structure.

The cardo structure is similar to the spiro structure, with “Cardo” meaning “hinge” or “ring” in Latin. Unlike the two-ring spiro structure, the cardo structure has only one ring connected to the cardo center, with the ring side chain and the main chain sharing a quaternary carbon atom [74]. Introducing the cardo structure into PI can retain excellent optical transparency and thermal properties. Our research group [75] prepared a non- functionalized cardo-type dianhydride from cyclopentanone and phenol to balance the comprehensive performance while reducing monomer synthesis costs, promoting the industrialization of cardo-type PI materials. Using 1,1-bis(4-(3,4-dicarboxy benzoyloxy)phenyl)cyclopentane (BDPCP) and aromatic 3,3′,4,4′-benzophenone tetracarboxylic dianhydride and four aromatic diamines, we prepared a series of cardo-type PI films (Figure 13). The alicyclic cyclopentane structure disrupted polymer chain conjugation and blocked charge transfer pathways, resulting in high optical transparency with λ_0_ ranging from 375 to 395 nm and transmittance between 72.6% and 83.5% at 500 nm. Additionally, the steric hindrance effect of the cyclopentane ring effectively enhanced the rigidity of PI chains, with T_d5_ in nitrogen ranging from 431 to 441 °C and T_g_ values between 217–271 °C, exhibiting good thermal properties.

**Figure 14:**
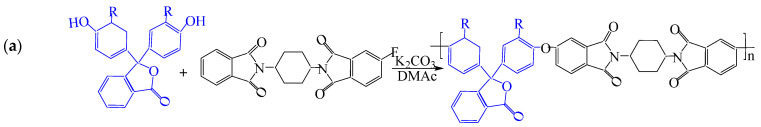

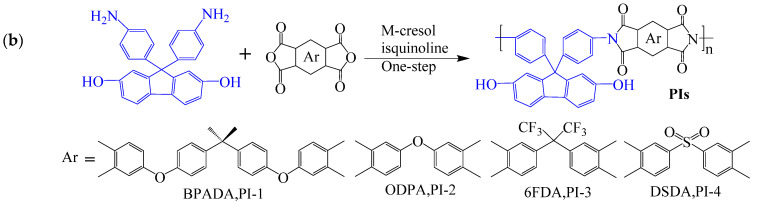
(**a**) Synthesis of PIs; (**b**) synthetic scheme of PIs with cardo structure.

Tang et al. [76] prepared two series of new CHDA-based transparent cardo-type PI films using cardo phthalein and 1,4-bis(4-fluorophthalimide) cyclohexane (BFCH) as monomers (Figure 14a). The study suggests that the large-volume structure of cardo phthalein could retain PI rigidity and extend chain spacing, effectively inhibiting conjugation and reducing CTC formation. The introduction of alicyclic structures disrupted the conjugation effect of PI, further inhibiting CTC formation. The synergistic effect of the two structures resulted in PI films with good optical transparency, with λ_0_ less than 355 nm and transmittance between 75% and 84% at 400 nm. Additionally, the series of PI films exhibited T_d5_ in nitrogen above 415 °C, increasing with the trans configuration ratio of CHDA in the polymer chain, showing good thermal stability.

In recent years, the preparation of hydroxyl-containing cardo-type PIs has been reported. Wu et al. [77] prepared a series of hydroxyl-containing cardo-type PI films (PI–1~PI–4) using diamine monomer AHF and dianhydrides BPADA, ODPA, 6 FDA, and DSDA (Figure 14b). The study suggested that the typical twisted non-coplanar cardo structure in AHF disrupted chain regularity, weakening intermolecular interactions, reducing CT effects, and improving optical transparency of PI films. Except for the highly rigid PI–4 film, PI–1~PI–3 films exhibited transmittance above 82% at 500 nm, with λ_0_ of 366 nm, 365 nm, and 349 nm, respectively. Additionally, strong hydrogen bonding between hydroxyl groups and the rigidity of the cardo structure enhanced PI thermal stability. The series of films exhibited T_d5%_ above 480 °C and T_g_ values above 410 °C in nitrogen. The hydroxyl groups on PI chains could also interact with F^−^, making the hydroxyl-containing cardo-type PI in dimethyl sulfoxide (DMSO) solution visibly yellow–green, with the solution color changing significantly with increasing F^−^ concentration, exhibiting high selectivity and sensitivity to fluoride ions. This study provided a simple and convenient method for visual detection of F^−^ using hydroxyl-containing cardo-type PI films, expanding their application in chemical sensors. 

### 3.4. End Group Optimization Strategies

PI film coloration also results from the thermal imidization process at the final stage of film preparation, where amino end groups of PAA molecular chains produce colored products under thermal and chemical imidization. To explore the detailed mechanism of PI film coloration, Yue et al. [78] prepared PI films using ODA and BPDA. They found that amino end groups formed –N=O, –NO_2_, and other chromophores absorbing blue light (435–480 nm), causing yellowing of the film. The –N=O also underwent electrophilic condensation with amino end groups of PI chains, forming colored azo structures, further coloring the PI film. Additionally, anhydride end groups of PI chains underwent electrophilic arrangement reactions with diamine segments during heating, forming colored anthraquinone structures, significantly affecting the transmittance of PI film materials in the UV–visible light region.

To effectively reduce the adverse effects of colored anthraquinone structures on film coloration, special groups can be used to cap PI molecular chains, regulating PI’s optical transparency. Su et al. [79] cooperated to use 3,3′-sulfonyl-dianiline (APS) with electron-withdrawing –SO_2_– and 2,2′-bis(trifluoromethyl) benzidine (TFMB) with steric hindrance –CF_3_ and BAPP to prepare three PI films with BPDA and 6 FDA, respectively, using 6 FDA and flexible ethylene glycol (EG) as end-capping agents to cap anhydride end groups of PI molecular chains. The strong electron-withdrawing ability of 6 FDA reduced electron density of adjacent atoms, effectively inhibiting electrophilic reactions forming colored anthraquinone at the diamine ends. Combining APS with –SO_2_– and TFMB with –CF_3_ electron-withdrawing effects and the large steric hindrance effect of these diamines further prevented electrophilic reaction of anhydride end groups, effectively suppressing anthraquinone formation and reducing PI film coloration. The study also found that EG and 6 FDA end-capping agents could reduce Rayleigh scattering from nanoscale ordered structures, significantly improving transparency in the visible light region. The PI films exhibited λ_0_ between 334 and 388 nm, with maximum transmittance in the UV–visible range up to 98.2% for PITFt-F/E film prepared with TFMB/6 FDA main chain and 6 FDA and EG as end-capping agents.

With the increasing demand for efficient and rapid heat dissipation in consumer electronics, the thermal conductivity PI film industry has developed rapidly. However, traditional PI films have low intrinsic thermal conductivity (λ), failing to meet the requirements of flexible displays, foldable screens, and flexible wearable devices, resulting in poor operating stability and service lifespan [80]. Liquid crystal PI films (LC-PI) with micro-order characteristics of liquid crystal molecules provide an effective way to improve λ. However, when the temperature drops to room temperature, the liquid crystal structure of LC-PI disappears, restoring the disordered state of micro molecular chains, unable to maintain the intrinsic thermal conductivity of liquid crystal films.

**Figure 15:**
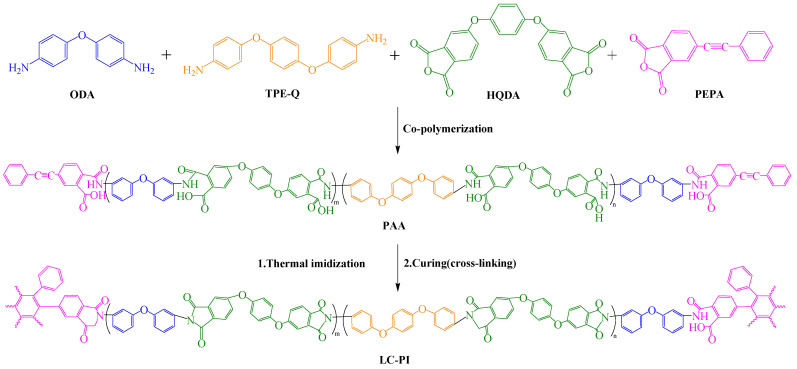
Synthetic scheme of liquid–crystalline PI.

Ruan et al. [81] found that using 4-phenylethynylphthalic anhydride (PEPA) as an end-capping agent for PI could solidify the ordered degree of PI molecular chains through chemical cross-linking reactions of phenylethynyl groups at high temperatures, maintaining the liquid crystal structure of the film (Figure 15). Even when the temperature returns to room temperature, the micro-order of LC-PIIV molecular chains remains high. Due to the highly ordered molecular chains and thermal vibrations, phonon scattering between molecular chains was small during heat transfer, increasing λ value, making LC-PIIV films exhibit excellent thermal conductivity. Applying this LC-PI film as packaging material for flexible electronic devices can effectively dissipate chip heat, reducing chip temperature and solving the heat accumulation problem of flexible electronic devices. Additionally, compared to the non-capped N-PI film with n(ODA)/n(TPE-Q) of 1:3, LC-PIIV films also exhibited excellent mechanical properties and thermal properties, with tensile strength, tensile modulus, and T_g_ reaching 119.0 MPa, 2.1 GPa, and 262.4 °C, respectively. This cross-linked film has potential applications in high-heat fields such as highly integrated flexible electronics.

## 4. Practical Applications and Future Development Directions

High-performance polyimide (PI), aramid (AR), and nylon (PA) serve as the foundation and frontier polymers of the new round of technological revolution and industrial transformation, playing essential roles in strategic emerging material fields such as new energy, sophisticated components and devices, green environmental protection, and biotechnology. Among them, PI materials are known as the “problem solvers” and “golden films” for their excellent chemical resistance, radiation resistance, superior thermal properties, and mechanical properties. PI materials exhibit high application value in aerospace, sophisticated electronic components, semiconductors, and other cutting-edge fields, with broad application prospects in solar cells [82,83,84], gas separation membranes [85,86,87], liquid crystal display devices [88,89], and more. However, the high-performance PI film market is mainly dominated by DuPont in the United States, Ube Industries, Kaneka and Mitsubishi Gas in Japan, and SKP in South Korea [90]. Large-scale production of advanced PI film products continues to be in urgent need of supporting the development of related industries.

Introducing rigid structures into the molecular main chain can further enhance the rigidity of PI materials, improving their thermal and mechanical properties. Increasing backbone flexibility can effectively improve the optical properties of PI films at the expense of thermal stability to some extent. Molecular design optimization from three aspects, side chains, non-coplanar structures, and end groups, can synergistically improve the optical and thermal performance of PI films. However, the optimized design, synthesis technology, and cost-effectiveness of these new functional PIs still restrict their broad applications in emerging fields such as flexible display substrates, aerospace engine materials, and high-frequency communication materials. Table 1 lists the characteristics analysis and practical application fields of molecular structure optimization strategies of PIs.

Future Molecular Design Directions:

(1) Nanoparticle doping and organic polymer blending modification: Nanoparticle doping modification to prepare PI hybrid materials can provide PI with superior comprehensive optical and thermal performance. Our research team introduced non-coplanar bulky alicyclic structural units with electron-donating effects to reduce the rigidity of PI molecules and increase the electron cloud density of the carbonyl and nitrogen atoms on the imide ring. We incorporated inorganic nanoparticles with low resistivity, corona resistance, and thermal stability such as TiO_2_ and BaTiO_3_. Meanwhile, organic polymers such as nanocellulose (CNC) [91], polyperfluoroethylene propylene (FEP) [92], and polymethylmethacrylate (PMMA) [93] can be blended with PIs to alter the interfacial polarization and molecular arrangement, suppress space charges, and expand the application of PIs in high-performance capacitors, optoelectronic devices, etc. We compared the modification principles, advantages, disadvantages, and applications of hybrid modification and molecular structure optimization, as shown in Table 2 [94,95,96,97,98].

(2) Intrinsic microporous PI membranes: Extensive studies should be addressed to further explore intrinsic microporous PI polymers, study the relationship between the microstructure of PI films and gas permeability selectivity, design special diamine or dianhydride monomers to control micropore size, distribution, and gas interactions in PI films, decrease ordered aggregation states, improve H_2_ selectivity in PI gas separation membranes, extend the applications to hydrogen production fields, and achieve greater economic, social, and ecological benefits. Microporous PI membranes for bio-medical application deserve in-depth research, as PIs have exhibited desired bio-compatibility.
(3) Photosensitive polyimides (PSPIs): PSPIs combine excellent thermal stability, mechanical properties, and photosensitivity, and are primarily used in photoresists and electronic packaging. PSPI production is based on photosensitive PAA, whose imidization could be properly accelerated by promoters under optical irradiation. Chang et al. [99] developed a series of negative-type PSPIs based on PAA and a photobase generator (PBG), finding that the rigidity and transparency of the PAA/PI backbone play an important role in the sensitivity and contrast of PSPI. Future research should focus on introducing suitable photosensitive groups, optimizing preparation processes, and re-pondering PSPI mechanisms in the revolutionary chip fabrication process, expanding PSPI applications in upgraded microelectronics packaging, optoelectronic packaging, and other fields.
(4) Machine learning (ML) applications: Data-driven machine learning (ML) can quickly predict material properties, accelerating the screening of new materials and molecular structure design [100]. ML’s generative inverse design networks can take target functions or properties as input, searching corresponding molecular structures, conducting quickly high-throughput screening of PI films with multiple properties, and predicting new PI films with superior comprehensive optical and thermal performance.
(5) Film surface metal-coating technology: Metal coating technology on film surfaces can provide electrical pathways for components packaged on the film surface, expanding applications in optics, electronics, solar energy, and micropatterning fields. Common methods include copper-clad laminate (CCL) and dense, adhesive metal films generated on PI films through sputtering and other dry processes. However, CCL technology is challenging to apply in fine wearable and portable devices, and sputtering processes are costly process. Zhong et al. [101] proposed a new technology for fabricating high-precision copper conductive micropatterns on PI films by directly preparing conductive copper micropatterns through electroless plating, without expensive lithography equipment and complex vacuum environments, reducing PI coating costs. Future research should explore PI film surface structure modification and metal-coating technology to meet the needs for miniaturization and multifunctionality in wearable and portable devices.

## 5. Conclusions

This review highlights the latest research advancements in collectively enhancing the optical and thermal performance of PI films and proposes various optimization strategies from molecular design to practical applications. PI films, known for their excellent chemical resistance, radiation resistance, thermal properties, and mechanical properties, show significant application value in advanced fields such as aerospace, sophisticated electronic components, and semiconductors. However, balancing optical transparency and thermal performance in traditional PI films remains a challenge. This review summarizes various strategies to enhance the optical and thermal performance of PI films through main chain, side chain, non-coplanar structures, and end group optimization, and Table 3 presents a brief summary of the typical monomer designed and optimized by PI molecular structures in recent years.

By introducing rigid and flexible structures into the molecular main chain, optimizing side chains with fluorinated substituents and large side groups, and applying non-coplanar structures such as spiro and cardo structures, the optical transparency and thermal properties of PI films can be significantly improved. Additionally, end group optimization strategies reduce coloration during PI film preparation, further enhancing optical performance. These strategies improve the comprehensive performance of PI films and expand their applications in flexible displays, solar cells, and high-performance electronic devices.

Future development should be focused on the technical and cost-reduction challenges of functional PI films. Emerging research directions, including nanoparticle doping and organic polymer blending modification, intrinsic microporous PI membranes, photosensitive polyimides, machine learning-assisted molecular design, and film surface metal-coating technology, provide new pathways for further enhancing the optical and thermal performance of PI films. These technological breakthroughs will help PI films achieve widespread application in more high-tech fields.

In summary, systematic molecular design and structural optimization have been able to and should continue to significantly improve the optical and thermal performance of PI films, showing great potential in broadening PI’s application prospects. Future research should continue to focus on the relationship between material structure and performance, exploring more efficient and economical preparation methods to promote the deep integration of PI films into emerging technology and industry.

## Figures and Tables

**Table 1:** Characteristics analysis and practical application fields of PI molecular structure optimization strategies.

Optimization Strategy	Optimized Structure	Strengths	Weaknesses	Representative Application	Ref.
Main-chain optimization	Rigid structure	Resonance of coplanar imide ring with aromatic rings, strong electron delocalization and interchain CT, excellent thermal and mechanical properties	Poor processability, low optical transparency	Solar cell flexible substrate, flexible copper clad laminate, electronic device heat dissipation	[23,24,25]
	Hydrogen bond	Non-covalent interaction, amide and hydroxyl as two main hydrogen bonding groups, increasing the chain rigidity while maintaining the optical transparency.	Tend to dissociate under harsh working condition, potentially leading to instability in the performance of PI films	Substrate for flexible organic light-emitting diode (OLED) displays, FPC matrix film	[26,27,28,29,30,31,32]
	Flexible structure	Decreased mainchain coplanarity, weakened interchain CT, increased processibility, and improved optical transparency	Formation of insoluble nylon salt for alicyclic diamine, reduced thermal and mechanical performance	Plastic substrates for color filters, optical compensating films, optical fibers, wave guide, and optical lens	[35,36,37,38,39,40,41,42,43,44,45,46,47]
Side-chain optimization	Fluorinated groups	Steric bulkiness and electron-withdrawing capability, reduced interchain CT, hydrophobic and nonpolarized properties leading to low water affinity and low dielectric constant	Difficult to achieve fluorinated PI monomers, high cost of fluorinated monomers	Flexible display panel, optical wave communication device, image display device, liquid crystal orientation layer	[51,52,53]
	Tert-butyl	Increased steric hindrance, reduced chain packing, inhibited electron flow in the molecular chain, improved processibility and optical transparency	Complicated synthesis of new dianhydride and diamine monomer containing pendent t-butyl	Liquid crystal display, gas separation membrane	[53,54,55,56,57,58,59,60,61,62]
Functional backbone structural optimization	Spiro structure	Highly twisted structure, deformed polymer chain, enlarged interchain space, improved optical transparency, improved gas permeability, maintained thermal properties	Easy to plasticize and age	High-temperature-resistant gas separation membrane	[68,69,70,71,72,73]
	Cardo structure	Non-coplanar structure, weakened interchain CT, improved optical transparency, maintained thermal performances	Reduced chemical stability	Thin-film optoelectronic devices, chemical sensors	[75,76,77]
Terminal group optimization	Functional capping structure	Strong electron-withdrawing groups capping structure leading to improved optical transparency, reactive capping agent leading to cross-linked and ordered macromolecular chains	Bringing new chromophore, reduced CT energy gap, needing to balance curing temperature and liquid crystal transition temperature	Flexible display substrate, high-temperature flexible electronics field	[78,79,80,81]

**Table 2:** Comparison of two PI modification optimization strategies: nanoparticle hybrid modification and molecular structure optimization.

Strategy	Hybridization			Structural Optimization			
Classification	Organic Polymer	Inorganic Particle	Organic- Inorganic Hybrid Particles	Main-Chain Optimization	Side-Chain Optimization	Non-Coplanar Optimization	Terminal Groups Optimization
Strengths	Good compatibility with PI, robust mechanical properties of composites	Low resistivity, good corona resistance, and thermal stability	Rigidity, excellent thermal stability, and adjustable dielectric properties	Regulating the properties of PIs by modifying the rigidity and flexibility of polymer backbone	Enhancing steric hindrance, inhibiting electron delocation	Reducing the degree of coplanarity of PI molecular chains and optical absorption	Adjusting thermal stability and optical transparency of PI by controlling chain packing
Weaknesses	Reduced thermal stability when excessive doping	Poor compatibility and dispersion	Complicated preparation process and high cost	Difficult to balance the optical and thermal properties	Delicacy in balanced structural design	Lack of novel non-coplanar structure design	Introducing new chromophore, unmatched curing temperature
Application	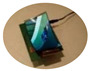 OLED	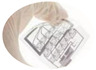 Flexible device	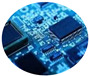 Integrated circuit package	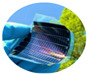 Solar cell	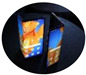 Flexible substrate	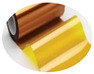 Gas separation membrane	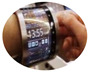 Flexible display devices

**Table 3:** Typical monomers for PI molecular structure design and optimization.

Monomer Structure	Full Name	Abbreviation	Ref.
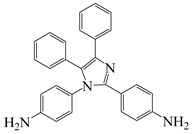	4,4′-(4,5-diphenyl-1 H-imidazole-1,2-diyl)dianiline	DIMA	[25]
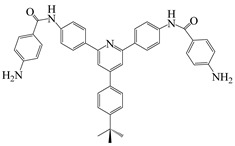	N,N-((4-(4-(tertbutyl)phenyl)pyridine-2,6-diyl)bis(1,4-phenylene))bis(4-aminobenzamide)	NTPA	[30]
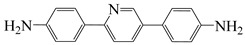	2,5-bis(4-aminophenyl)pyridine	PRD	[32]
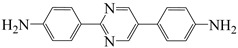	2,5-bis(4-aminophenyl)pyrimidine	PRM	[32]
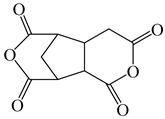	2,3,5-Tricarboxycyclopentylacetic dianhydride	TCA-AH	[35]
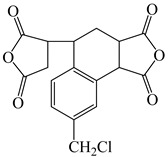	3,4-Dicarboxy-1,2,3,4-tetrahydro-6-chloro-methyl-1-naphthalene succinic dianhydride	CMTDA	[35]
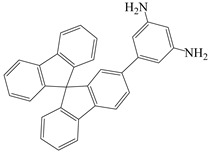	2-(3,5-diaminobenzene)-9,9′-spirobifluorene	35 DABSBF	[36]
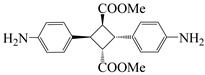	4,4′-diamino-α-truxilic acid	4 ATA	[36]
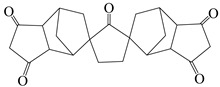	Norbornane-2-spiro-α-cyclopentanone-α′-spiro-2′-norbornane-5,5′,6,6′-tetracarboxylicdianhy-dride	CPODA	[36]
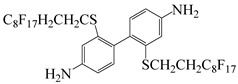	2,2′bis((1 H,1 H,2 H,2 Hperfluorodecyl)thio)[1,1′-biphenyl]4,4′-diamine	BPFBD	[36]
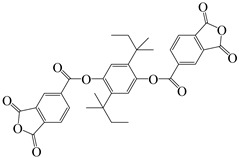	2,5-di-tert-butylhydroquinone	25 DBHQ	[36]
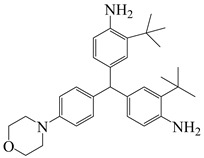	3,3′-di(tert-butyl)-4,4′-diaminodiphenyl-4′-morpholinophenylmethane	TAMPM	[36]
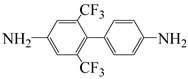	2,6-bis(trifluoromethyl)benzidine	TFMT	[37]
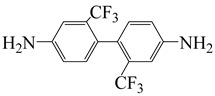	2,2′-bis(trifluoromethyl)benzidine	TFDB	[37]
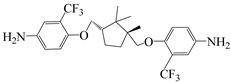	(+)-cis-1,3-bis(4-amino-2(trifluoromethyl)phenoxylmethylene)-1,2,2-trimethylcyclopentane	BAFMT	[54]
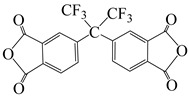	2,2′-bis-(3,4-dicarboxyphenyl)hexafluoro-propane dianhydride	6 FDA	[55]
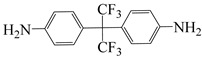	4,4′-(hexafluoroisopropylidene)dianiline	6 FDAM	[55]
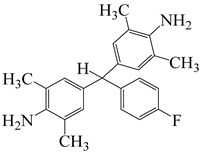	α,α-bis(4-amino-3,5-dimethylphenyl)-1-(4′-fluorophenyl)methane	BAFM	[56]
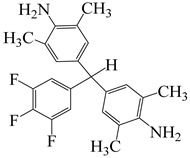	α,α-bis(4-amino-3,5-dimethyl-phenyl)-1-(3′,4′,5′-trifluorophenyl)methane	BATFM	[56]
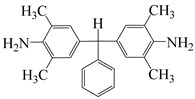	α,α-bis(4-amino-3,5-dimethylphenyl)-1-phenylmethane	BAPM	[56]
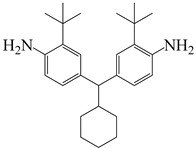	4,4′-(cyclohexylmethylene)bis(2-(tert-butyl)aniline)	CHMBTBA	[61]
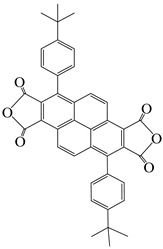	3,8-di(4-tert-butylphenyl)pyrene-1,2,6,7-tetracarboxylic dianhydride	DPt	[62]
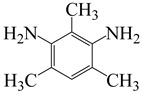	2,4,6-trimethyl-m-phenylenediamine	TMPD	[62]
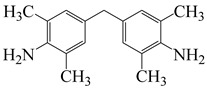	4,4′-methylenebis(2,6-dimethylaniline)	MBDAM	[62]
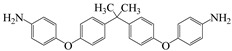	4,4′-((propane-2,2-diylbis(4,1-phenylene))bis(oxy))dianiline	BAPHF	[62]
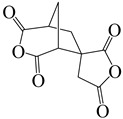	(1′R,3 S,5′S)-spiro[furan-3(2 H),6′-[3]oxabicyclo [3.2.1]octane]-2,2′,4′,5(4 H)-tetrone	DAn	[71]
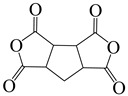	1,2,3,4-cyclopentanetetra carboxylic dianhydride	CPDA	[71]
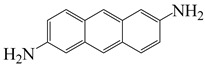	2,6-diaminoanthracene	AnDA	[72]
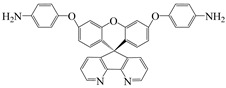	2′,7′-bis(4-aminophenoxy)-spiro(4,5-diazafluoren-9,9′-xanthene)	PSDA	[73]
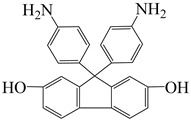	9,9-bis(4-aminophenyl)-2,7-dihydroxy-fluorene	AHF	[77]
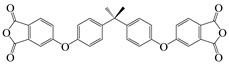	4,4′-(4,4′-Isopropylidenediphenoxy) diphthalic anhydride	BPADA	[77]
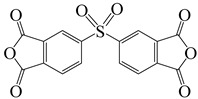	3,3′,4,4′-diphenylsulfonetetracarboxylic dianhydride	DSDA	[77]
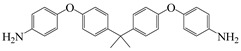	4,4′-((propane-2,2-diylbis(4,1-phenylene))bis(oxy))dianiline	BAPP	[79]
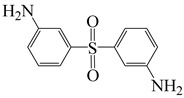	3,3′-sulfonyldianiline	APS	[79]

## Data Availability

No new data were created or analyzed in this study.
